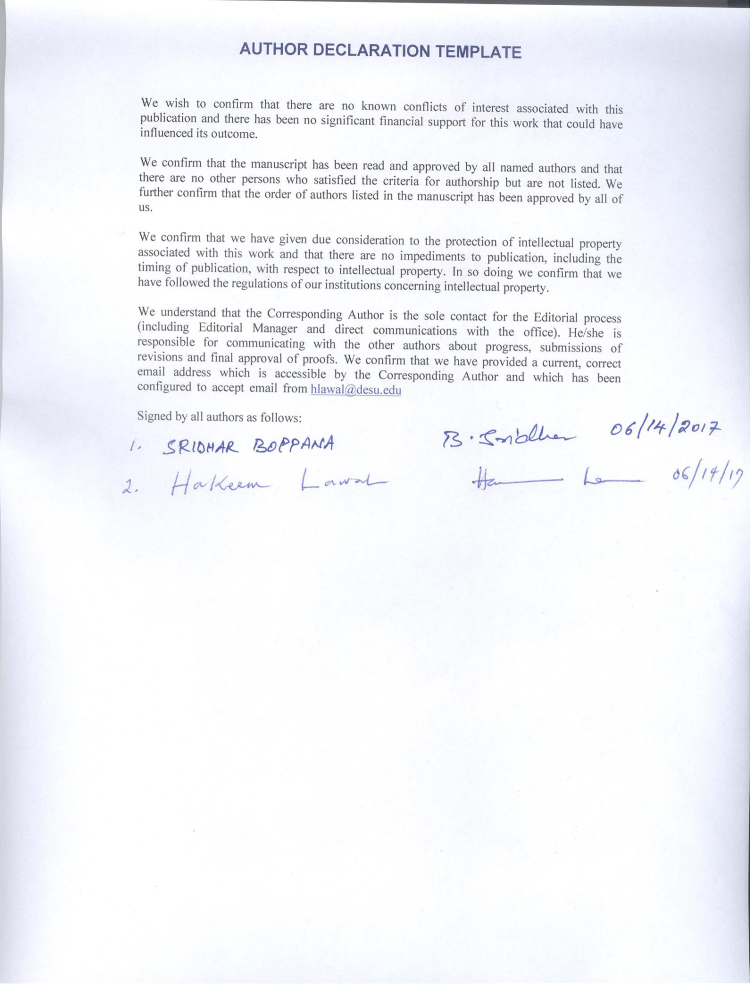# Data on the specificity of an antibody to *Drosophila* vesicular acetylcholine transporter

**DOI:** 10.1016/j.dib.2017.09.008

**Published:** 2017-09-23

**Authors:** Sridhar Boppana, Hakeem O. Lawal

**Affiliations:** Neuroscience Program, Department of Biological Sciences, Delaware State University, 1200 N. Dupont Highway, Dover, DE 19901, USA

## Abstract

The role of the vesicular acetylcholine transporter (VAChT) in the regulation of cholinergic neurotransmission has not been fully elucidated. Here we sought to develop a tool for studying vesicular acetylcholine transporter function, and we present data on the validation of our new anti-VAChT antibody. We show that the immunoreactivity of the antibody is not due to an artifact of secondary antibody staining, and we present two additional validation data. First, the peptide epitope used to generate the antibody is able to block the binding of the anti-VAChT antibody *in vivo*. Further, RNA interference (RNAi) -mediated knockdown of VAChT function in cholinergic neurons drastically reduces anti-VAChT staining in those constructs. Additional evidence for the antibody functionality is presented in our research article on the subject (Boppana et al., 2017) [1].

**Specifications Table**

**Table:** 

Subject area	*Neurobiology*
More specific subject area	*Cellular Neuroscience, Synaptic Neurotransmission*
Type of data	*Figures*
How data was acquired	*Confocal microscopy using the Olympus FV10i (Center Valley, PA)*
Data format	*Processed TIFF Image*
Experimental factors	*N/A*
Experimental features	*Samples were prepared using a protocol for whole-mount immunohistochemistry as described previously**[2]**. Briefly, Drosophila brains were dissected and immunostained as described elsewhere**[1]**. Imaging was then carried out using confocal microscopy as mentioned above.*
Data source location	*N/A*
Data accessibility	*The data are with this article*

**Value of the data**

• Describes a key resource for dissecting cholinergic neuron function in specialized neural circuits in the *Drosophila* nervous system.
• The data may be relevant for current efforts to map the circuitry of neurotransmitter systems within the brain.
• Describes two independent RNAi knockdown fly constructs of VAChT in cholinergic neurons that can be used to test the biological function of VAChT in those neurons *in vivo.*
• The *in vivo* peptide titration experimental procedure could be useful in validating other antibody reagents besides those developed against vesicular transporters.

## 1 Data

The data described here shows the specificity of the *Drosophila* anti-VAChT antibody. In *Drosophila* whole mount immunohistochemical preparations, we present data indicating that the VAChT antibody staining is not due to a secondary antibody artifact. Moreover, we show a dramatic reduction in VAChT immunostaining in fly constructs with an RNAi knockdown of VAChT expression in cholinergic neurons. Importantly, we present data on the titration of the VAChT antibody against the peptide that was used to generate the antibody. All of these data are available within this article.

## 2 Experimental design, materials and methods

### 2.1 Drosophila strains

**Fig. 1:**
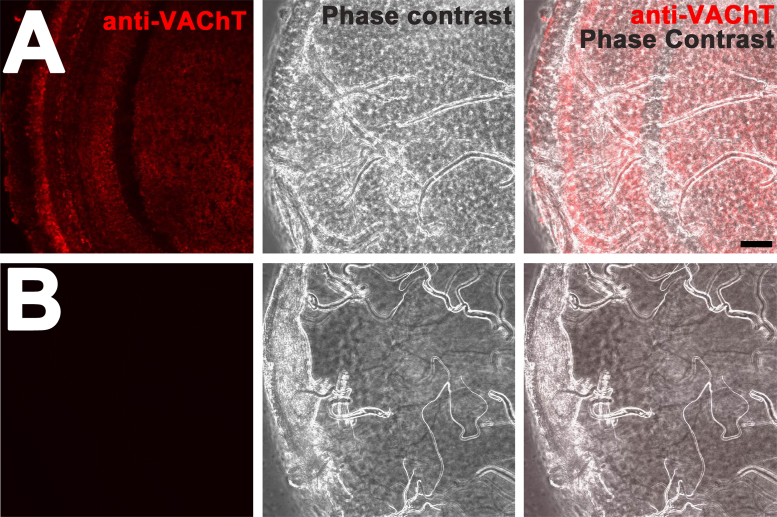
VAChT staining is not due to a secondary antibody artifact. The new antibody was raised against a 15-amino acid sequence near the C terminal region of VAChT [1] (A) VAChT staining (red) is readily observed in optic lobes of adult Drosophila whole mount preparations. Phase contrast image provides context about the tissue structure and anatomy. (B) No detectible signal is observed in the secondary antibody-only staining. Phase contrast image shows the structure and anatomy of the tissue. Each micrograph represents a single optical confocal slice. Scale bar, 20 um. Images were acquired under identical confocal microscope capture settings, post-processing was performed using identical brightness/contrast settings.

**Fig. 2:**
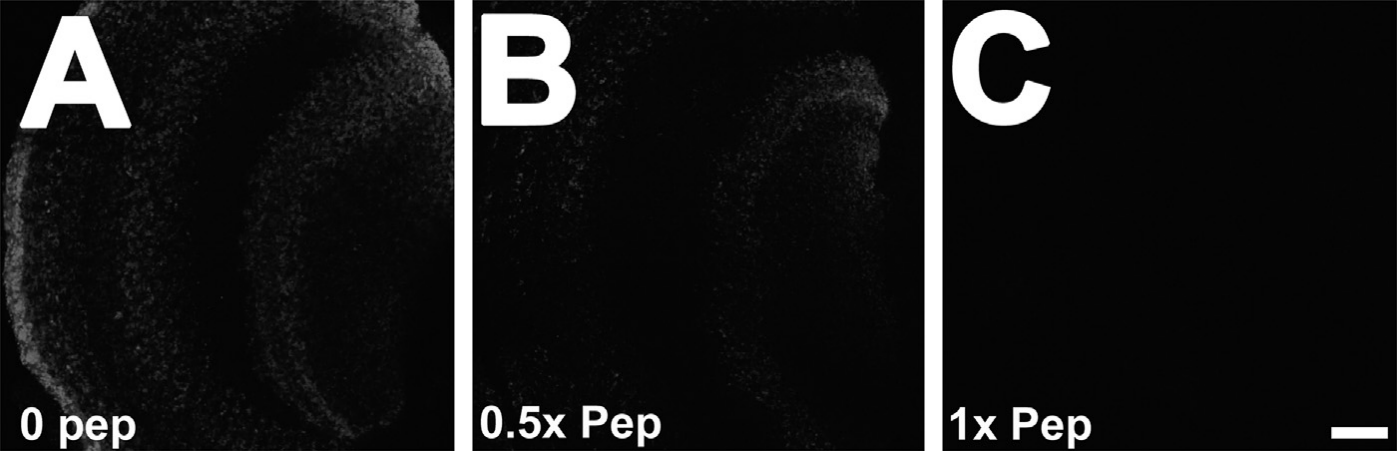
Increased concentration of VAChT epitope/peptide diminishes VAChT staining. (A) VAChT staining (1:1000) is performed in the presence of 0 peptide and VAChT staining pattern is readily observed in the optic lobe. (B) At 0.5x peptide concentration (and a 1:1000 anti-VAChT concentration), there is a reduction in VAChT staining. (C) VAChT staining is barely detectible when 1x peptide is mixed with anti-VAChT antibody. Each micrograph represents a single optical confocal slice. Scale bar, 20 um. Images were acquired under identical confocal microscope capture settings, post processing was performed using identical brightness/contrast settings.

**Fig. 3:**
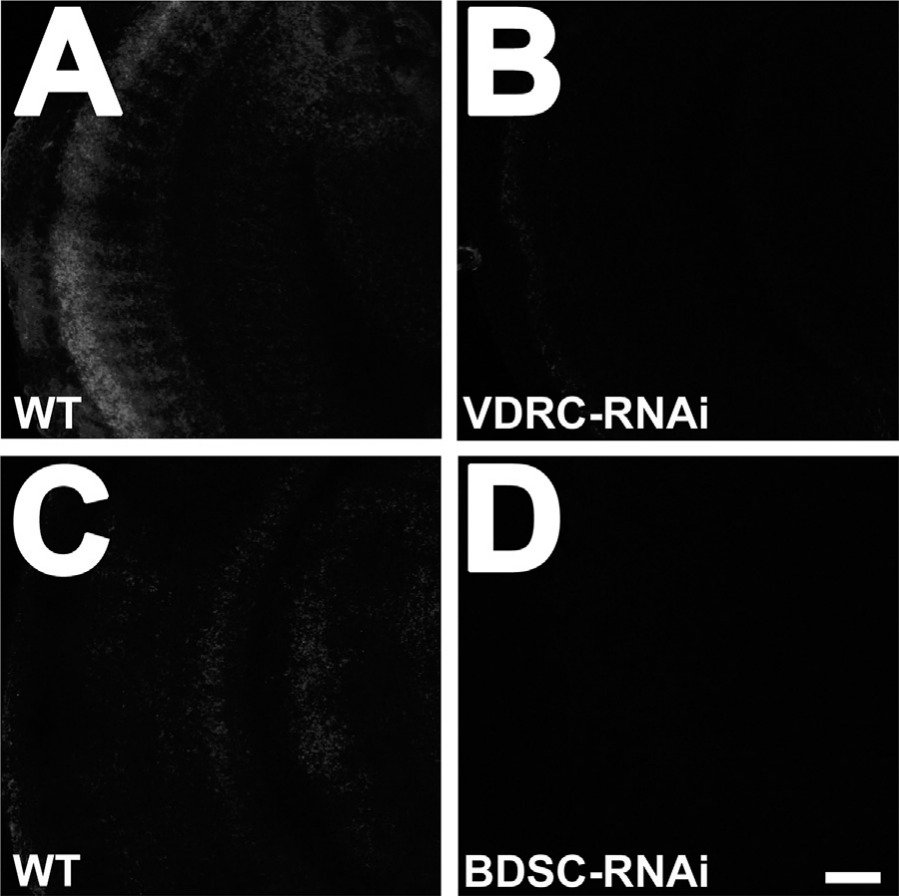
RNAi knockdown of VAChT in cholinergic neurons reduced VAChT expression. (A) VAChT staining in optic lobes of ChAT-Gal4,UAS-GFP/UAS-VAChT RNAi (Vienna Drosophila Research Center VDRC) (designated as “VDRC-RNAi” in the figure) shows a strong reduction in VAChT signal compared to ChAT-Gal4,UAS-GFP/+ control (designated as “WT” in the figure) (B) The RNAi experiment was performed using an independent UAS-RNAi construct from the Bloomington Drosophila Research Center (BDSC) (designated as “BDSC-RNAi” in the figure) and a reduction in staining is also observed compared to ChAT-Gal4,UAS-GFP/+ (designated as “WT” in the figure). Note that baseline signal intensity is lower in B relative to A. Each micrograph represents a single optical confocal slice. Scale bar, 20 um. Images were acquired under identical confocal microscope capture settings, post processing was performed using identical brightness/contrast settings. The data are representative of three independent experiments.

Fly stocks *w*^*1118*^*CS15*, *w; ChAT-Gal4,UAS-GFP* (BDSC # 6793), *yv; +; UAS-VAChT-RNAi* (BDSC # 27684), and *w*^*1118*^*, +, UAS-VAChT-RNAi* (VDRC # 40918) were used for the immunohistochemical assays. For the RNAi experiments, *w; ChAT-Gal4;UAS-GFP* was crossed into *UAS-VAChT-RNAi* and the progeny was stained for VAChT. The progeny of a cross between *ChAT-Gal4, UAS-GFP* and *w*^*1118*^*CS15* was used as control. All stocks were maintained on standard corn meal media and raised at 25 °C at a relative humidity of 50% (Fig. 1, Fig. 2, Fig. 3).

### 2.2 Peptide-titration immunohistochemistry

Wildtype fly brains were isolated and fixed in PFA as described elsewhere [1]. The epitope used as an antigen to generate VAChT antibody was diluted in the VAChT antibody to a 1:1 M ratio (see [1]). This peptide-antibody mixture or VAChT antibody without any peptide (control) was allowed to incubate with fly brains overnight at 4 °C. Brains were stained, mounted, and imaged as described below.

### 2.3 Immunostaining and confocal microscopy

Immunostaining and confocal microscopy were performed as described previously, see [1] and [2].

## Figures and Tables

**Figure:**